# Can CT or MRI volumetry substitute scintigraphy in living kidney donor evaluation? A systematic review

**DOI:** 10.1007/s00345-024-05024-y

**Published:** 2024-06-21

**Authors:** Alicia López-Abad, Thomas Prudhomme, Alessio Pecoraro, Romain Boissier, Muhammet Irfan Dönmez, Alberto Piana, Beatriz Bañuelos Marco, Mario Belmonte, Sergio Serni, Riccardo Campi, Angelo Territo

**Affiliations:** 1 Department of Urology, Virgen de La Arrixaca University Hospital, Murcia, Spain; 2 Unit of Urological Robotic Surgery and Renal Transplantation, Careggi Hospital, University of Florence, Viale San Luca, 50134 Florence, Italy; 3 Department of Urology, Kidney Transplantation and Andrology, Toulouse Rangueil University Hospital, Toulouse, France; 4 Department of Urology and Renal Transplantation, Aix-Marseille University, La Conception University Hospital, Marseille, France; 5 Department of Urology, Istanbul University Istanbul Faculty of Medicine, Istanbul, Turkey; 6 Department of Oncology, Division of Urology, University of Turin, Turin, Italy; 7 Department of Urology, Kidney Transplantation and Reconstructive Urology, Hospital Universitario Clinico San Carlos, Madrid, Spain; 8 Department of Experimental and Clinical Medicine, University of Florence, 50134 Florence, Italy; 9 Department of Urology, Fundació Puigvert, Autonoma University of Barcelona, Barcelona, Spain

**Keywords:** Nephrectomy, Kidney donor, Renal function, Living kidney transplantation, Computed tomography, Scintigraphy, Magnetic resonance imaging

## Abstract

**Background:**

Current potential living kidney donor’s assessment includes functional and anatomical evaluation. Scintigraphy is recommended in some cases and some centers include this test in the donor’s protocol. Recent studies advocate for the avoidance of this test as CT or MRI volumetry showed to accurately assess donor’s renal function.

**Objective:**

To summarize scientific evidence on image tests for pre-donation and/or post-nephrectomy renal function evaluation.

**Evidence acquisition:**

This review followed the guidelines set by the European Association of Urology and adhered to PRISMA 2020 recommendations. The protocol was registered in PROSPERO on 10th December 2022 (ID: CRD42022379273).

**Evidence synthesis:**

Twenty-one studies met the inclusion criteria after thorough screening and eligibility assessment. According to QUADAS-2, patient selection and flow/timing domains showed a predominant low risk of bias.

The correlation between split renal function (SRF) using CT and scintigraphy varied from weak (*r* = 0.21) to remarkably strong (*r* = 0.949). Bland–Altman agreement demonstrated moderate to excellent results, with mean differences ranging from -0.06% to 1.76%. The correlation between split renal volume (CT) and estimated glomerular filtration rate (eGFR) at 6 months or 1 year after nephrectomy showed a moderate correlation, with coefficients ranging from 0.708 to 0.83.

The correlation between SRF (MRI) and renal scintigraphy reported a moderate correlation, with correlation coefficients of 0.58 and 0.84. MRI and scintigraphy displayed a good agreement, with a 66% agreement observed and mean differences of ± 0.3%.

**Conclusions:**

Despite study heterogeneity, MRI or CT-based renal volumetry appears promising compared to scintigraphy, with favorable correlations and agreement.

**Supplementary Information:**

The online version contains supplementary material available at 10.1007/s00345-024-05024-y.

## Introduction

Living donor kidney transplantation (LDKT) is the most valuable source of organs for kidney transplantation (KT) worldwide. It has been promoted during the last decades as a crucial strategy to increase the number of organs and try to reach the increasing demand. In addition, LDKT has demonstrated superior outcomes in terms of organ survival, morbidity, and mortality compared to cadaveric donor KT [1]. This could be related to the shorter waiting time (or even the avoidance of dialysis) and the better quality of the organs with reduced ischemia time.

Living kidney donor needs a multidisciplinary evaluation to ensure that neither the donor nor the recipient assume excessive risks with the interventions. Historically, preoperative donor work-up includes renal function (creatinine clearance) and anatomical assessment (CT angiography). However, the SRF (defined as the proportion of total RF contributed by each kidney) should be measured by combining 51Cr EDTA and 99mTc DMSA in concrete occasions, such as when there is a size disparity between the two kidneys, the renal function (RF) is close to the acceptable threshold for the donation, or there is anatomical abnormality or complexity [2]. Moreover, to assess the potential donor’s RF carefully, some centers protocols include performing both imaging tests (CT and scintigraphy). In contrast, recent studies advocate for the avoidance of renal scintigraphy and instead suggest CT or MRI volumetry, as non-invasive, easy-to-perform, resource-saving, and reliable alternatives. Different studies indicate that pre-donation renal volumetry could accurately assess SRF and post-donation kidney function (PDKF). Cohort studies in healthy patients with an average glomerular filtration rate have demonstrated that renal volume is very similar in both kidneys [3–6]. Despite these promising results, there is still lack of evidence regarding the most appropriate preoperative differential RF assessment for kidney donor candidates.

To fill this gap, we conducted a systematic review of the available scientific evidence pertaining to imaging modalities for pre-donation RF evaluation of potential donors and/or for the PDKF evaluation.

## Material and methods

### Evidence acquisition

This systematic review was conducted following the principles highlighted by the European Association of Urology guidelines [7] and following the Preferred Reporting Items for Systematic Reviews and Meta-analyses (PRISMA) 2020 recommendations [8]. The protocol was registered in the International Prospective Register of Ongoing Systematic Reviews (PROSPERO; http://www.crd.york.ac.uk/prospero) on 10th December 2022 (ID:CRD42022379273).

### Search strategy

A systematic literature screening was conducted by two authors (ALA and TP) using the Cochrane Library, MEDLINE/Pubmed and Embase databases. The search was limited to articles published in the English language between January 2000 and January 2023. The search strategy incorporated both free-text and MeSH terms (Appendix 1). Furthermore, the reference lists of the initially selected articles were manually scrutinized to identify additional studies of relevance. The final list of selected articles underwent comprehensive review and approval by all co-authors.

### Inclusion and exclusion criteria

A specific population (P), intervention (I), comparator (C), outcome (O), and study design (S) (PICOS) framework was assessed to define the study eligibility.

The PICOS framework for this review was as follows:

-(P): adult (age > 18 years) kidney donor candidates.
- (I): living donor nephrectomy.
- (C): comparative studies.
- (O): donor’s SRF and/or PDKF measured by eGFR.
- (S): prospective or retrospective studies published in English-language between January 2000 and January 2023, reporting pre-donation and/or PDKF comparison between an image test (volumetry calculation) and the current gold standard, scintigraphy.

### Study selection and data extraction

Two members of the research team (ALA and TP) independently conducted a systematic search of the previous mentioned databases, following the PICOS framework as listed. The screening of titles and abstracts was performed using Rayyan (Rayyan Systems, Cambridge,MA, USA). Subsequently, the full texts of the remaining studies were examined by all co-authors after the exclusion process.

Data extraction was carried out using a pre-defined spreadsheet developed in advance and managed by two members of the team (ALA and TP). In instances where discrepancies arose regarding study selection or data extraction, resolution was achieved by another author (RC).

The following data were extracted for each study:

- Study identification: authors, publication year, country.
- Methods: study design, study period, predicted outcome (evaluate SRF, predict PDKF, eGFR), eGFR formula used, follow-up timing.
- Participant characteristics: number of patients, number of events (pre-donation RF, PDKF, SRF), inclusion and exclusion criteria.
- Imaging test characteristics:
  - Imaging test type:CT or MRI
  - CT: technique, contrast medium used, slice thickness, volume calculation
  - Scintigraphy: nuclear GFR tracer
- Statistical analysis data: agreement (between tests, interobserver agreement), correlation, interobserver concordance.

### Risk of bias (RoB) assessment

RoB assessment was performed independently by two authors (ALA and TP) using the Quality Assessment tool for Diagnostic Accuracy Studies (QUADAS-2) [9]. QUADAS-2 tool was used to assess the RoB over four domains, patient selection, index test(s), reference standard and flow and timing. The disagreement was solved by a third party (RC).

### Data synthesis

The methodological and clinical heterogeneity of the included studies implied that meta-analysis was inappropriate. Therefore, a narrative synthesis of the data was performed. The primary outcomes of interest pertained to the comparison of SRF between the different tests. Additionally, secondary outcomes focused on the evaluation of PDKF.

## Evidence synthesis

### Literature search and study characteristics

**Fig. 1:**
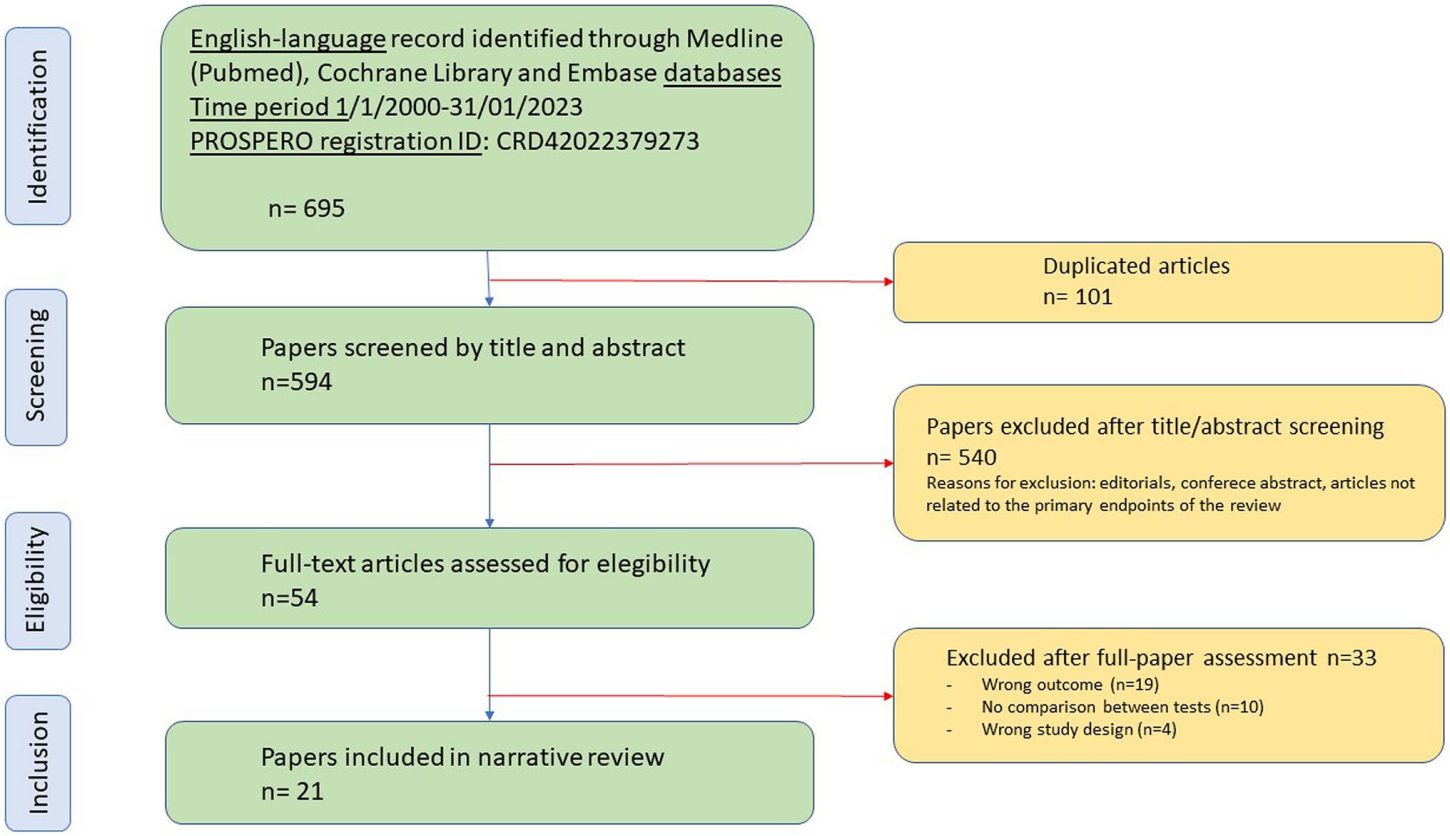
Flowchart showing the literature search and systematic review process according to the Preferred Reporting Items for Systematic Reviews and Meta-analysis (PRISMA) statement recommendations

The literature search initially included 695 papers. After screening and eligibility assessment, twenty-one studies met the inclusion criteria (Fig. 1).

Only three of the studies included were prospective design [3, 10, 11]. The samples sizes varied considerably, ranging from 12 patients [12] to 835 patients [13]. Furthermore, the timeframe for the included cohorts varied widely, ranging from 1997–2001 [14] to 2017–2020 [11]. A comprehensive breakdown of the main characteristics of the included studies is provided in Table 1.

**Table 1:** Overview of the main characteristics of the included studies

Authors	Study period	Patients	Outcome	Study design	Tests to compare	Timing of the outcome after surgery	Renal function pre and postdonation	Scintigraphy tracer	Computed tomography characteristics	Agreement	Correlation	Additional statistical analyses
Nilsson et al. [14]	1997–2001	27	Evaluate SRF	Retrospective cohort—Single-centre	CT vs. scintigraphy	–	–	MAG3	Technique: CTA Thickness: 2 mmVolume calculation: area x thickness	–	*r* = 0.9	–
Kato et al. [28]	2004–2007	28	Evaluate SRF	–	CT vs. scintigraphy	–	–	DMSA	Technique: MDCT Thickness: 5 mm Volume calculation: 3D reconstruction	Bland–Altman −0.2% (−3.5 to 3%)	*r* = 0.93	–
Summerlin et al. [24]	2005–2006	173	Evaluate SRF	Retrospective cohort—Single-centre	CT vs. scintigraphy	–	–	–	Technique: CTA Thickness: 2–3 mm Volume calculation: 3D reconstruction	Bland–Altman: −0.06% (−6.820 to 6.720%)	*r* = 0.61	–
Artunc et al. [10]	2007–2009	21	Evaluate SRF	Prospective cohort-Single-centre	MRI vs. Scintigraphy	–	–	DTPA	–	Plot	*r* = 0.84	–
Soga et al. [26]	2005–2010	38	Evaluate SRF	Retrospective cohort—Single-centre	CT vs. scintigraphy	< 12 months	–	MAG3 or DTPA	Technique: MDTC Thickness: —Volume calculation: Semiautomated volume; attenuation capacity; modified ellipsoid; parenchymal area; lenght x width; lenght	Bland–Altman modified ellipsoid bias −0.2% (−1.1 to 0.7%)	*r* = 0.84 (modified ellipsoid)	Interobserver agreement of CT-based methods: modified ellipsoid 0.1 (−0.8–1)
Patankar et al. [12]	2010–2011	12	Evaluate SRF and predict RPKF	Retrospective cohort—Single-centre	CT vs. scintigraphy	12 months	Preoperative eGFR 102 ± 11 mL/min/1.73m^2^	DMSA	Technique: CTA Thickness: —Volume calculation: area x thickness	Bland–Altman 1.29 ± 1.65	*R*^2^ = 0.22 (no significant)	Correlation between postoperative eGFR at 1 year and Scintigraphy: *R*^2^ = 0.69 CT *R*^2^ = 0.74
Halleck et al. [21]	2005–2011	144	Estimate or measure GFR	Retrospective cohort—Single-centre	CT vs. scintigraphy	6 months	Predonation eGFR (CG) 106 ± 27 mL/min/1.73m^2^. Postdonation eGFR (CG) 75 ± 20 mL/min/1.73m^2^	DTPA or MAG3	Technique: MDCT Thickness: 0.5–1 mm Volume calculation: 3D reconstruction	–	*r* = 0.93 Right kidney *r* = 0.66 Left kidney *r* = 0.62	Correlation of predonation SRF of the preserved kidney with postdonation eGFR (CG) of the donor at 1 year for MAG3 (*r* = 0.73), ROI (*r* = 0.75), MELV (*r* = 0.75) and RCV (*r* = 0.77)
Diez et al. [6]	2006–2011	190 (65)	SRF	Retrospective cohort—Single-centre	CT vs. scintigraphy	–	–	MAG3	Technique: MDCT Thickness: 1 mm Volume calculation: 3D reconstruction	0.65 +—3.46	*r* = 0.59	Correlation of post-donation eGFR at 6 months after donation and MAG3 *r* = 0.85; and vol *r* = 0.83
Wahba et al. [20]	2010–2014	101	Determine preoperative SRF and predict PKF	Retrospective cohort—Single-centre	CT vs. scintigraphy	–	Predonation eGFR: 109 ± 28 mL/min/1.73m^2^	MAG3	Technique: helical MDCT Thickness: 2 mm Volume calculation: modified ellipsoid	Bland–Altman SRF . ROI vs MAG 3 0.4% (−7.7% to 8.6%). MELV vs MAG3 0.4% (−8.9% to 9.7%) . RCV vs MAG3 0.8% (−9.1% to 10.7%)	*r* = 0.21	Correlation of predonation SRF of the preserved kidney with postdonation eGFR MAG3 *r* = 0.73 ROI *r* = 0.75 MELV *r* = 0.75 RCV *r* = 0.77
Weinberger et al. [4]	2012–2014	13	Evaluate SRF	Retrospective cohort—Single-centre	CT vs. scintigraphy	–	–	MAG3	Technique: MDCT Thickness: 0.5–0.65 mm Volume calculation: 3D reconstruction	Bland–Altman −3.9 to 5.8	Spearman rho = 0.76	–
Yanishi et al. [19]	–	35	Evaluate SRF	–	CT vs. scintigraphy	–	Preoperative eGFR 99.1 ± 17.2 mL/min/1.73m^2^	MAG3	Technique: MDCT Thickness: – Volume calculation: –	–	*r* = 0.714	Correlation eGFR and SRV after 1 year *r* = 0.708; and SFR-MAG3 *r* = 0.634
Yokoyama et al. [22]	2009–2013	46	Evaluate SFR	–	CT vs. scintigraphy	–	Preoperative creatinine clearance 117.1 ± 36.9 mL/min/1.73m^2^	MAG3	Technique: MDCT Thickness: —Volume calculation: 3D reconstruction	Bland–Altman 1.2% (−4.9 to 7.2%)	*r* = 0.441	–
Barbas et al. [23]	2009–2011	224	Determine SRF (*n* = 224). Predict RPKF (*n* = 88)	Retrospective cohort—Single-centre	CT vs. scintigraphy	6 months	Preoperative eGFR 95.1 ± 14.5 mL/min/1.73m^2^ Predicted postoperative eGFR—Scintigraphy 47.6 ± 8.1 mL/min/1.73m^2^—CT 47.5 ± 7.6 mL/min/1.73m^2^	DTPA	Technique: CTA Thickness: —Volume calculation: attenuation method	Agreement observed between tests 78.5%	SRF: Left kidney *r* = 0.51 Right kidney *r* = 0.52 RPKF: Left kidney *r* = 0.53 Right kidney *r* = 0.56	Kappa test: 0.25 Right kidney *R*^2^ = 26.2%; Left kidney *R*^2^ = 26.7% SRF > 10% scintigraphy 7.6% SRF > 10% in CT 19.20%
Lee et al. [17]	2013–2015	264	Evaluate predicted eGFR	–	CT vs. scintigraphy	6 months	Predonation eGFR 103.62 ± 20.09 mL/min/1.73m^2^	DTPA	Technique: MDTC Thickness: 1–3 mm Volume calculation: parenchyma x thickness	–	*r* = 0.949	Correlation of post-donation eGFR at 6 months after donation and DTPA *r* = 0,711; and vol *r* = 0,747 DTPA-eGFR AUC 0,858 (0,804–0,911 *p* = 0,000); Vol-eGFR AUC 0,878 (0,829–0,928 *p* = 0,000)
Mitsui et al. [16]	2009–2016	34	Evaluate SRF	Retrospective cohort—Single-centre	CT vs. scintigraphy	12 months	Preoperative eGFR 72 mL/min/1.73m^2^ (59.7–105.7) Postoperative eGFR 47.7 mL/min/1.73m^2^ (34.4–65.1)	MAG3	Technique: MDTC Thickness: 1 mm Volume calculation: 3D reconstruction	Bland–Altman Cortex −0,32% (−1.19 to 0.55%) Parenchyma −0.46% (−1.20 to 0.28%)	Cortex *r* = 0.921 Parenchyma *r* = 0.942	Correlation of post-donation eGFR at 12 months after donation and MAG3 *r* = 0.763 Cortex volume split *r* = 0.747 Parenchyma volume split *r* = 0.764
Lange et al. [15]	2010–2014	100	Predict post-donation kidney function	Retrospective cohort—Single-centre	MRI vs. Scintigraphy	3 years	Predonation eGFR: 99.13 ± 28.5 mL/min/1.73m^2^ SRF left kidney: 51.66 ± 3.96% SRF right kidney: 48.34 ± 3.96% Postdonation eGFR 69.78 ± 21.16 mL/min/1.73m^2^	MAG3	–	Agreement observed between tests: 66%	Total kidney function *r* = 0.6735 (*p* < 0.0001); Remaining kidney function *r* = 0.5877 (*p* < 0.0001)	–
Harper et al. [25]	2009–2019	248	Evaluate SRF and predict PKF	Retrospective cohort—Single-centre	CT vs. scintigraphy	> 6 months	Predonation eGFR 99 ± 20 mL/min/1.73m^2^ Postdonation eGFR 67 ± 22 mL/min/1.73m^2^	DTPA or MAG3	Technique: MDCT study (volume, modified ellipsoid and CC dimension) Thickness: —Volume calculation: 3d reconstruction; ellipsoid formula	Bland–Altman Renography vs CT volume: 0.76% (−7.6–9.15%)—Renography vs CC dimension: 0.44% (−7.06–7.94)—Renography vs modified ellipsoid: 1.01% (−8.38–10.42%)	–	SRF > 10% in scintigraphy 11% (26) SRF > 10% in CT: –
Lal et al. [11]	2017–2020	526	Evaluate SRF	Prospective cohort—Single-centre	CT vs. scintigraphy	–	–	DTPA	Technique: MDCT Thickness: 0.9–3 mm Volume calculation: attenuation method	Bland–Altman Left kidney: 0% (−2 to 2.1%) Right kidney: 0% (−2 to 2%)	Left kidney *r* = 0.953 Right kidney *r* = 0.955	Left kidney *R*^2^ = 90.8%, Right kidney *R*^2^ = 91.3%
Krumm et al. [3]	–	65	Evaluate SRF	Prospective cohort—Single-centre	MRI vs. Scintigraphy	–	–	DTPA	–	Bland–Altman: Left kidney 0.3% (−2.8 to 3.4%) Right kidney −0.3% (−3.4 to 2.9%)	Spearman correlation Left kidney: *p* = 0.19 (*p* = 0.13) Right kidney: −	Correlation MRI volume and MRN split renal function Spearman *p* = 0.24 (*p* = 0.05)
Eum et al. [13]	2005–2020	835	Evaluate SRF and predict PKF	Retrospective cohort—Single-centre	CT vs. scintigraphy	1 year	Predonation eGFR 100.01 ± 13.94 mL/min/1.73m^2^	DTPA	Technique: MDCT Thickness: 0.75 mm Volume calculation: Voxel-count method	Bland–Altman 1.76 ± 3.14 (−4.39 to 7.91)	*r* = 0.484 (*p* < 0.001)	Intraclass correlation coefficient (ICC) 0.647 Correlation of post-donation eGFR at 1 year with SRF: Vol% (*r* = 0.722), DTPA% (*r* = 0.692); *R*^2^ = 0.554
Almeida et al. [18]	2008–2017	193	Evaluate SRF	Retrospective cohort—Single-centre	CT vs. scintigraphy	1 year	Predonation eGFR (CG): 106.7 ± 21.9 mL/min/1.73m^2^ Postdonation eGFR at 12 months (CG) 74.7 ± 15.3 mL/min/1.73m^2^	DTPA	Technique: MDCT Thickness: 2.5 mm Volume calculation: voxel counting technique	Bland–Altman −1.2% (−8.51 to 6.11%) Agreement between techniques 55% (106)	–	Evaluation of percentage of remaining kidney volume *R*^2^ = 0.15 Kappa test 0.259 Correlation of post-donation eGFR at 12 months after donation and DTPA *R*^2^ = 0.52 CT *R*^2^ = 0.55

*CTA* CT angiography, *eGFR* estimated glomerular filtrate rate, *MDCT* multidetector or multiphase CT, *MELV* modified ellipsoid volume, *PKF* postdonation kidney function, *RCV* renal cortex volumetry, *ROI* smart region of interest, *RPKF* residual postoperative kidney function, *SRF* split renal function

Regarding the imaging modalities, three studies reported results from MRI [3, 10, 15] while eighteen from CT. As part of the inclusion criteria, all the studies compare their main outcomes with the gold standard, scintigraphy. Each included study underwent a correlation and agreement analysis between the two tests comparing SRF and/or PDKF. In addition, eight studies evaluate RF at *6 months or 1 year* after surgery [12, 13, 16–21].

### Functional and image tests outcomes

#### CT vs scintigraphy

##### SRF outcomes

The SRF correlation between CT and scintigraphy was assessed in 94.44% (17/18) of the studies. The correlation exhibited considerable variability, spanning from studies that reported a weak correlation (*r* = 0.21 [22]) to those demonstrating a remarkably strong correlation (*r* = 0.949 [17]). However, when considering the individual kidney comparison, the observed correlations improved significantly, ranging from 0.52 [23] to 0.955 [11].

Bland–Altman agreement was calculated in 72.22% (13/18) of the studies and found to be moderate to excellent, from a mean of −0.06% [24] to 1.76% [13]. Notably, Lal et al. demonstrated a perfect agreement of 0% in the individual comparison between the right and left kidney [11].

##### PDKF outcomes

PDKF was reported in 22.22% (4/18) of the studies [16, 18, 21, 25] but only one of them reported the predicted postoperative eGFR [23]. Furthermore, the correlation between split renal volume and eGFR at 6 months or 1 year after nephrectomy was assessed in 9 studies [6, 12, 13, 16–21]. The results showed a moderated level of correlation, with correlation coefficients ranging from 0.708 [19] to 0.83 [6] and coefficient of determination (R^2^) values spanning from 0.55 [18] to 0.74 [12]. This level of correlation is similar to that obtained when comparing scintigraphy-derived results to eGFR measurements (*r* from 0.634 to 0.85; *R*^2^ from 0.554 to 0.69).

#### MRI vs scintigraphy

##### SRF outcomes

The SRF correlation between MRI and renal scintigraphy was evaluated in 66.67% (2/3) of the comparative studies. The findings indicated a moderate correlation in both studies, with correlation coefficients of 0.58 and 0.84, respectively [10, 15].

Regarding the agreement analysis, different approaches were employed. One study utilized a plot method for evaluation, which proved challenging due to the absence of means and cut-offs provided by the authors [10]. In contrast, the other two studies applied the “agreement observed” between the two tests [15] and the Bland–Altman test [3]. These latter methods showed good agreement between MRI and scintigraphy, with a 66% agreement observed and mean differences of −0.3% in the right kidney and 0.3% in the left kidney, respectively.

##### PDKF outcomes

In the studies included, there was no assessment of correlation with PDKF.

### RoB assessment and generalizability

**Fig. 2:**
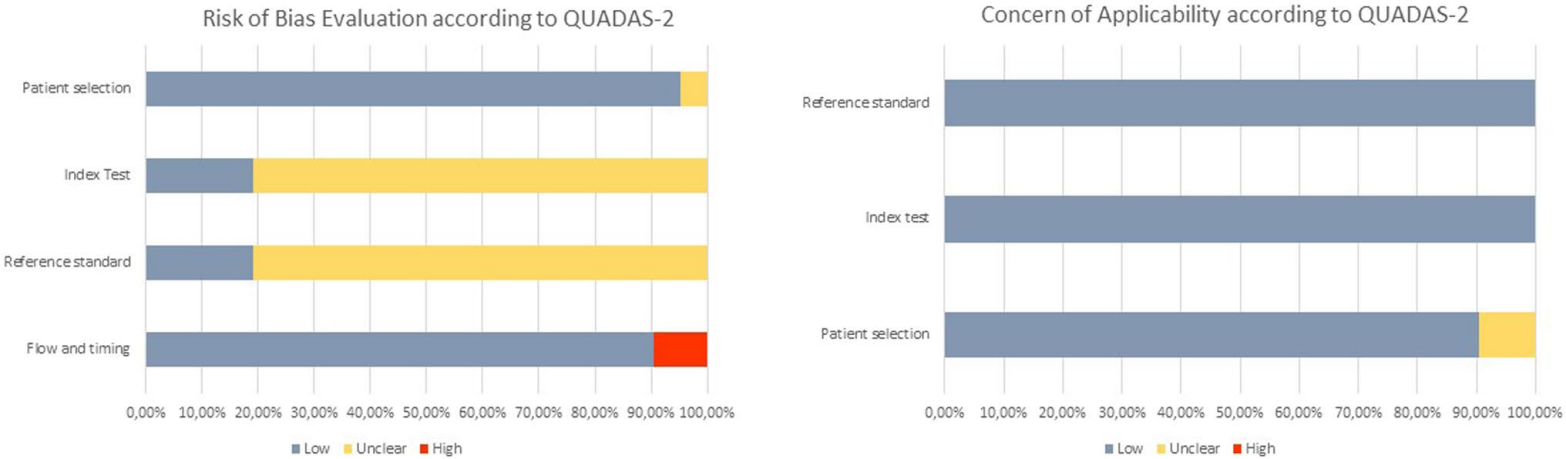
Overview of the overall risk of bias and applicability judgements for the studies included in the review according to the Quality Assessment of Diagnostic Accuracy Studies tool (QUADAS-2)

RoB was evaluated according to QUADAS-2 assessment tool, as shown in Fig. 2 and Supplementary Table 1. This tool revealed a predominant low RoB in patient selection and flow and timing domains and a majority low concern of applicability in all the domains studied. Only four studies [12, 24–26] indicated that the outcomes were blinded for the researchers or radiologists.

In three studies [18, 20, 21] the authors not only assessed living donors but also collected data on recipients. Nonetheless, the outcomes of donors and recipients were reported separately, allowing for a thorough and independent analysis of each group.

## Discussion

Donor’s evaluation is fundamental both for the receipt and the donor. In addition to anatomy and compatibility considerations, the potential donor’s assessment must prioritize the minimization of both short and long-term risks associated with nephrectomy. It is important to emphasize that donors are healthy individuals, willingly undergoing a nephrectomy, which is a high-risk surgical procedure [27], driven by altruistic intentions to "gift" their kidney, often to a relative.

There is an active debate on the donor’s optimal study pre-donation as recent studies support the option of performing only a volumetry test which can assess SRF and even PDKF instead of the actual (but not universalized) functional and anatomical evaluation or scintigraphy and CT.

Unfortunately, the availability of literature on SRF assessment through imaging tests remains limited, with only twenty-one studies being included in this review. Summarizing outcomes proved challenging due to the heterogeneity observed among the included studies, mainly concerning the methods employed for study design, scintigraphy tracer, CT characteristics, and statistical analysis. This heterogeneity rendered meta-analysis unfeasible. Furthermore, the absence of an established consensus makes direct comparisons arduous. Despite these challenges, the QUADAS-2 assessment revealed an acceptable RoB (Fig. 2).

Focusing on scintigraphy tracers, the studies included in this review used different agents, such as DTPA, MAG3, and DMSA, sometimes even within the same study [21, 26]. The choice of the radiopharmaceutical was dependent on the type of renal scan performed [30]. DTPA and MAG3 have proven useful in measuring GFR and evaluating flow through the pyelocalyceal system and bladder. These tracers are typically employed for dynamic imaging, while DMSA is primarily used for static imaging and cortical anatomy assessment, particularly for pathologies like renal ectopia or renal scarring [30]. There is currently lack of scientific evidence published on the optimal nuclear tracer for evaluating RF in a healthy population, such as potential kidney donor. Consequently, we can only speculate that the results obtained from different tracers yield similar outcomes. However, it is crucial to acknowledge that no concrete evidence supports this hypothesis.

In terms of CT characteristics, the studies reviewed employed various techniques (such as multidetector, conventional, and CT angiography), thickness (from 0.5 [4] to 5 mm [28]), and volume reconstruction calculation methods (automatic 3D reconstruction, area multiplied by thickness, modified ellipsoid…). This diversity complicates direct comparisons between studies. Regarding CT thickness, recent studies have yielded similar findings in assessing kidney volume, demonstrating low interobserver variability [29]. Focusing on volume reconstruction, there is currently no consensus on the method for the calculation of renal volumetry.

Our review revealed robust correlations between CT and scintigraphy, with good correlation coefficients ranging from 0.9 to 1 [14, 17, 21, 28], contrasting with Wahba et al. [20] lower correlations rate. Notably, these correlations held validity across studies with both small and large cohort populations, as evidenced in smaller studies [14, 28] compared those with larger cohort populations [17, 21]. Moreover, the correlation between MRI and scintigraphy also demonstrated favorable results, with a coefficient of 0.84 reported in the study conducted by Artunc et al.[10].

Regarding the agreement between tests, CT studies consistently showed the best correlations, as shown by the findings of different studies [11, 24, 26, 28]. Another small cohort study [3] also obtained excellent agreement between MRI and scintigraphy. Nevertheless, it is important to note that this level of agreement was not consistently observed in other MRI studies, particularly those with larger cohort populations [15].

The main limitation of this systematic review stems from the substantial heterogeneity observed in the literature due to the lack of robust evidence. However, these limitations serve as an initial catalyst for enhancing awareness and prompting improvements in study design, ultimately benefiting decision-making and diagnostic practices. Despite these challenges, our review demonstrated an acceptable RoB. Nevertheless, our systematic review provides valuable insights into the current state of evidence concerning imaging tests for assessing kidney function in potential donors.

## Conclusions

Living donor nephrectomy holds significant importance for clinicians, patients, their families, and transplant surgeons alike. However, the current state of evidence is marred by a notable lack of robust data, presenting a significant challenge to informed decision-making in clinical practice. Nevertheless, promising results have emerged from studies assessing renal volumetry through MRI or CT demonstrating favorable correlation and agreement compared to the established gold standard, scintigraphy. It is imperative to prioritize improvements in study design and initiate prospective registries dedicated to this area. Our systematic review represents the first step to acknowledge these limitations and pave the way for multidisciplinary prospective studies involving nephrologists and urologists.

## Supplementary Information

Below is the link to the electronic supplementary material.

Supplementary file1 (DOCX 75 KB)

Supplementary file2 (DOCX 17 KB)

Supplementary file3 (DOCX 19 KB)

## Data Availability

The authors confirm that the data supporting the findings of this study are available within the article and in the supplementary material.

## Abbreviations

eGFR: Estimated glomerular filtration rate
KT: Kidney transplantation
LDKT: Living donor kidney transplantation
PDKF: Post-donation kidney function
SRF: Split renal function
RF: Renal function
RoB: Risk of bias
