# Supplementary material for: Can CT or MRI volumetry substitute scintigraphy in living kidney donor evaluation? A systematic review
Source: World J Urol. 2024 Jun 21;42(1):382. doi: 10.1007/s00345-024-05024-y (PMC11192666; doi:10.1007/s00345-024-05024-y)
Supplement: Supplementary file 1 — Supplementary file1 (DOCX 75 KB) [file 345_2024_5024_MOESM1_ESM.docx]

| **Study** | **Risk of bias** | | | | **Applicability concerns** | | |
| --- | --- | --- | --- | --- | --- | --- | --- |
|  | **Patient selection** | **Index test** | **Reference standard** | **Flow and timing** | **Patient selection** | **Index test** | **Reference standard** |
| **Nilsson et al [14]** | 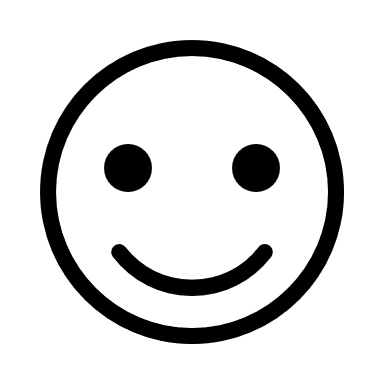 | 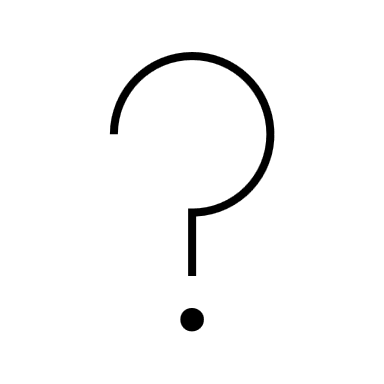 | 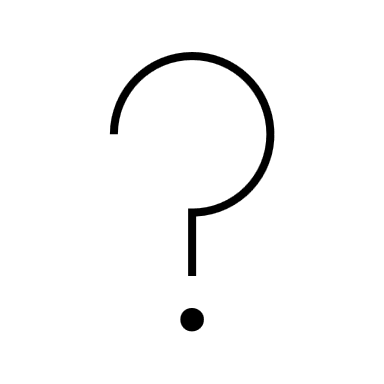 | 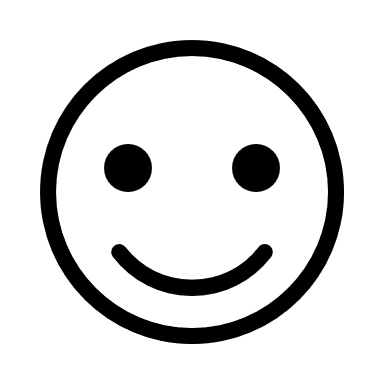 | 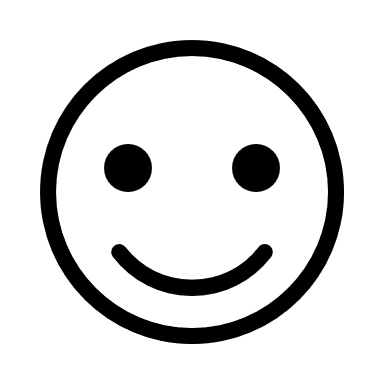 | 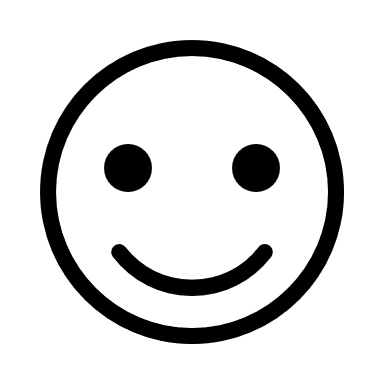 | 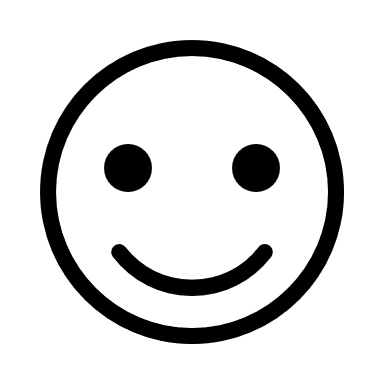 |
| **Kato et al [28]** | 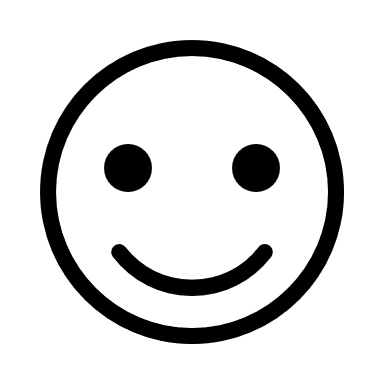 | 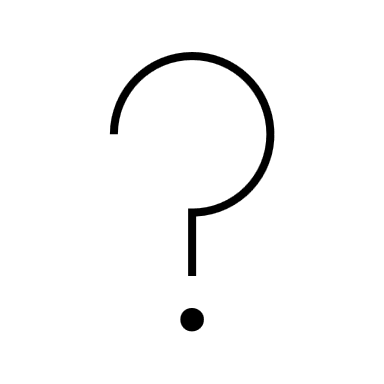 | 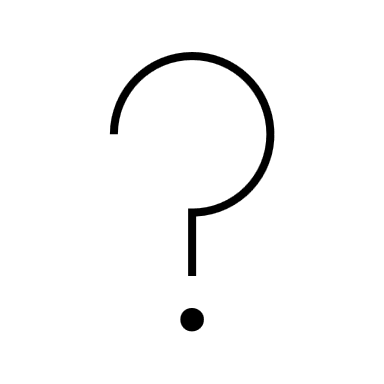 | 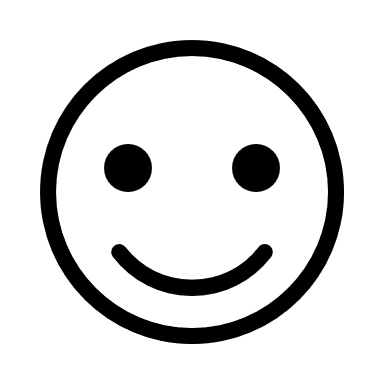 | 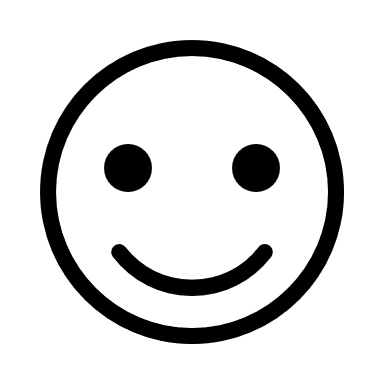 | 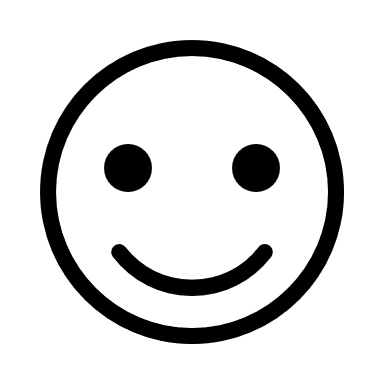 | 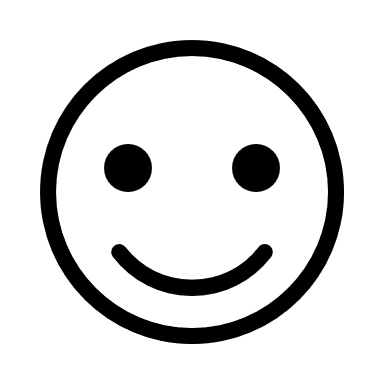 |
| **Summerlin et al [24]** | 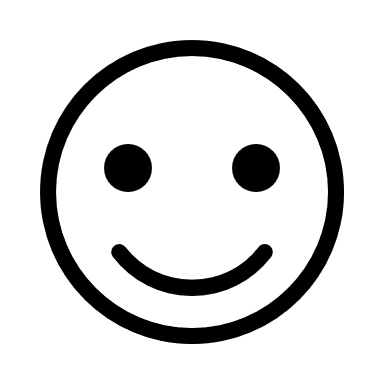 | 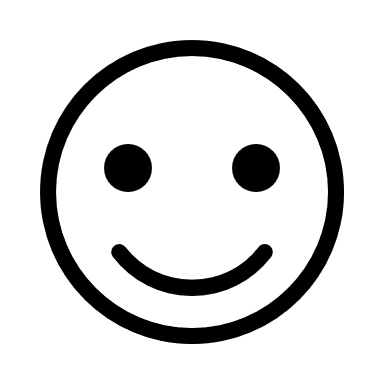 | 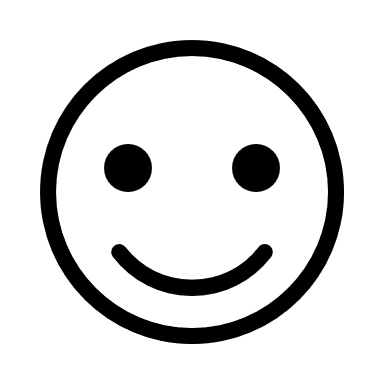 | 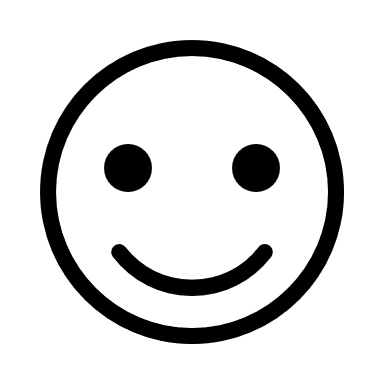 | 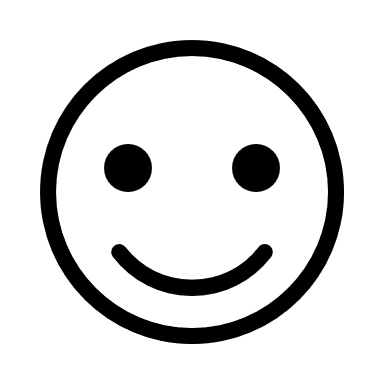 | 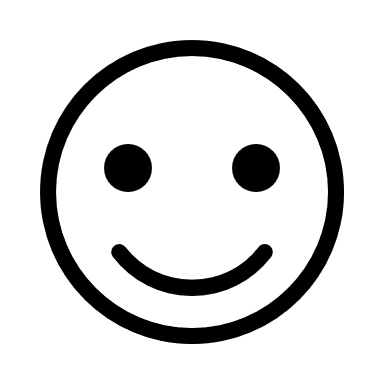 | 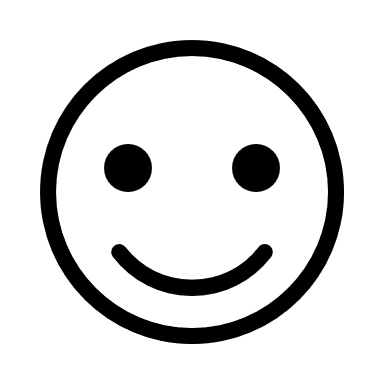 |
| **Artunc et al [10]** | 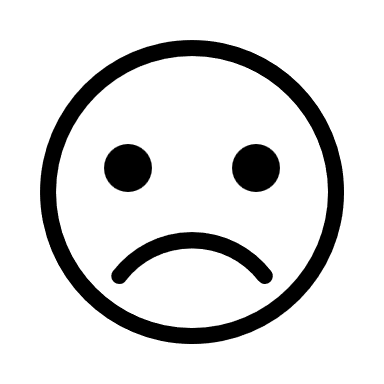 | 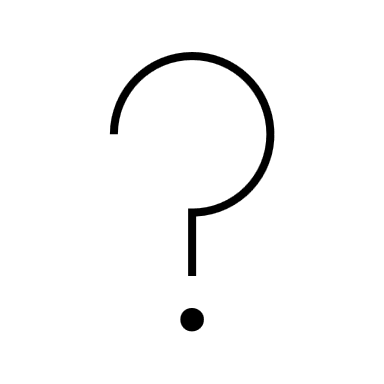 | 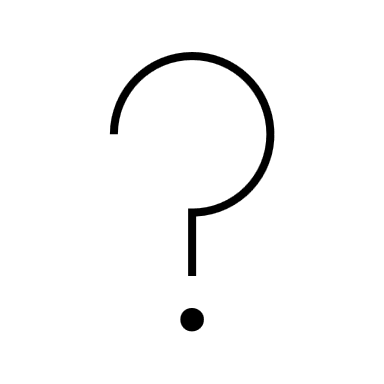 | 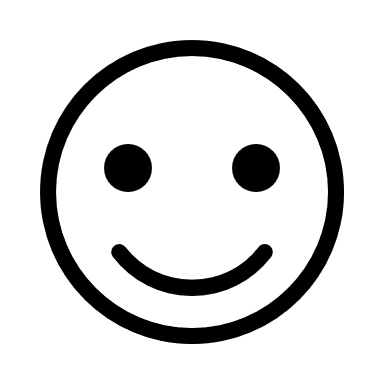 | 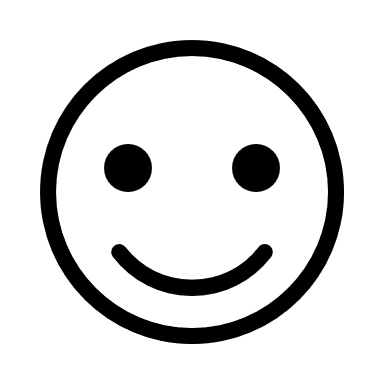 | 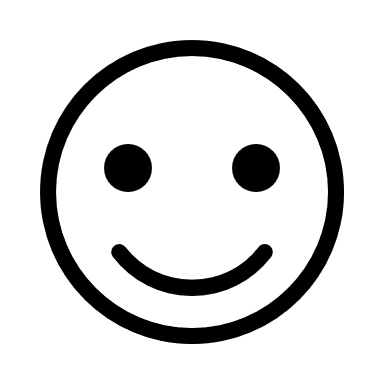 | 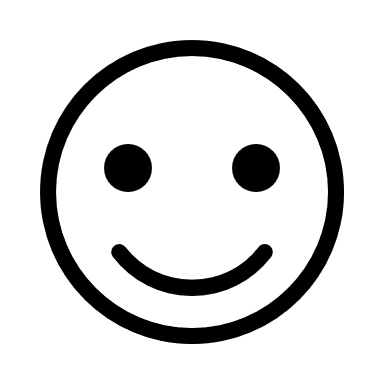 |
| **Soga et al [26]** | 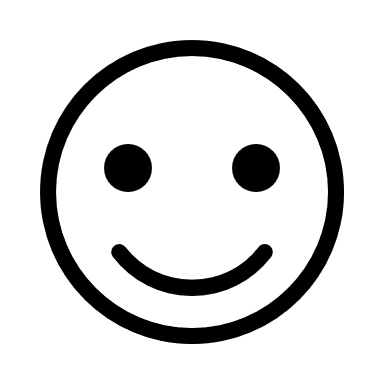 | 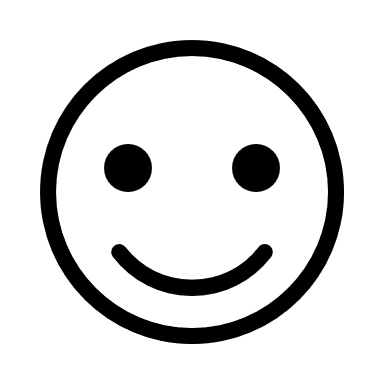 | 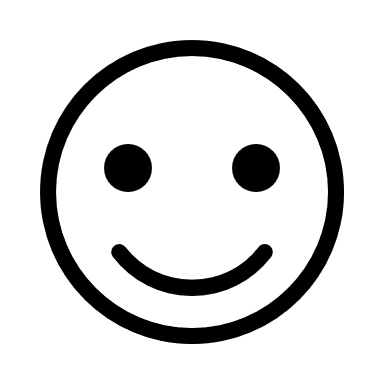 | 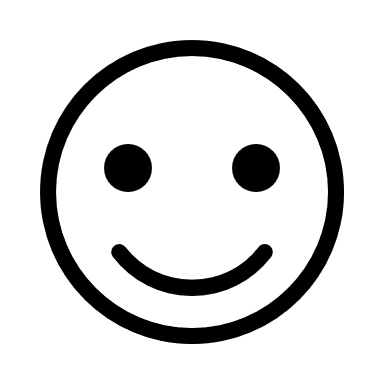 | 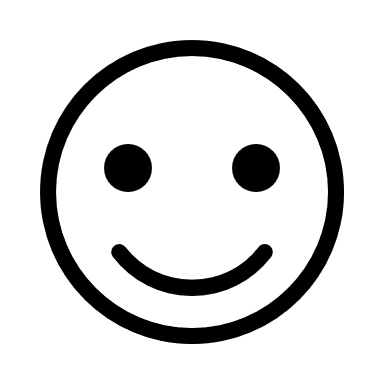 | 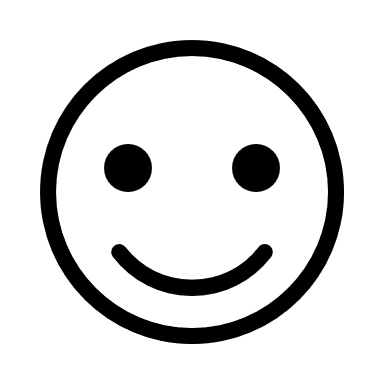 | 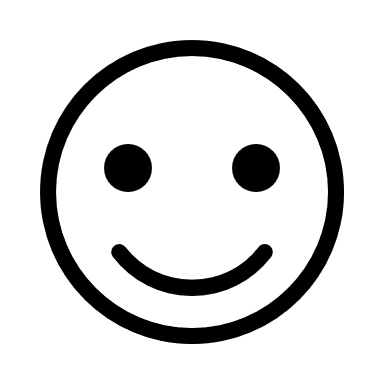 |
| **Patankar et al [12]** | 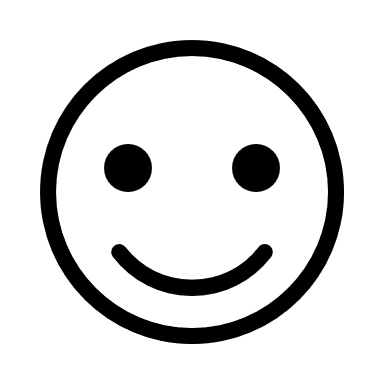 | 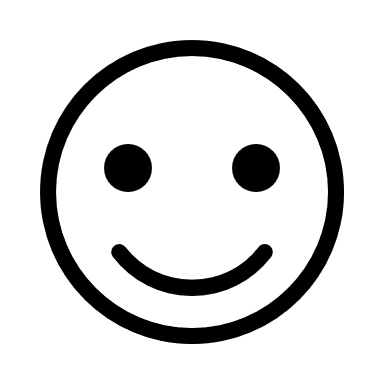 | 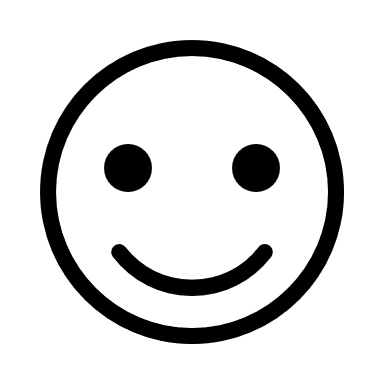 | 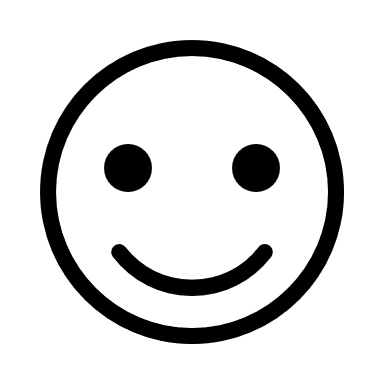 | 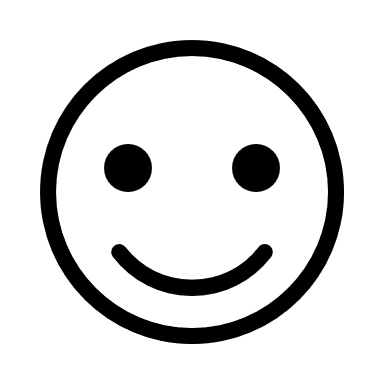 | 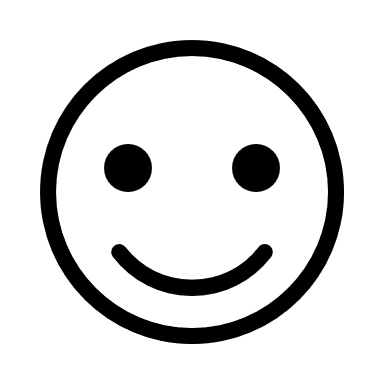 | 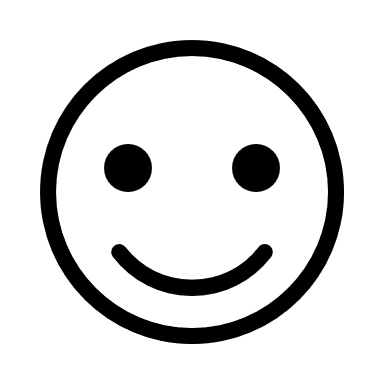 |
| **Halleck et al [21]** | 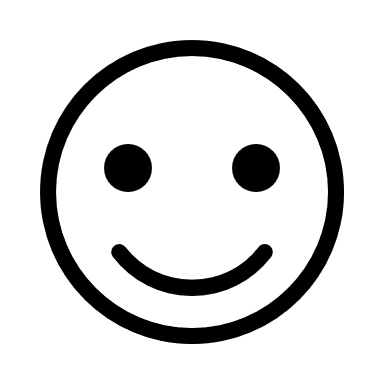 | 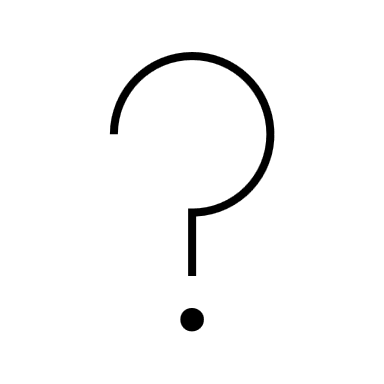 | 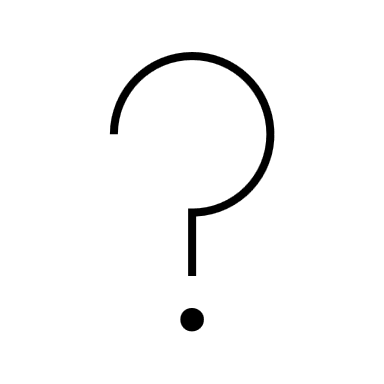 | 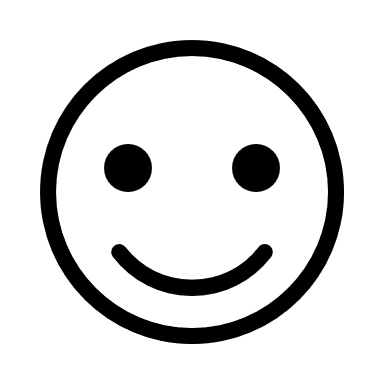 | 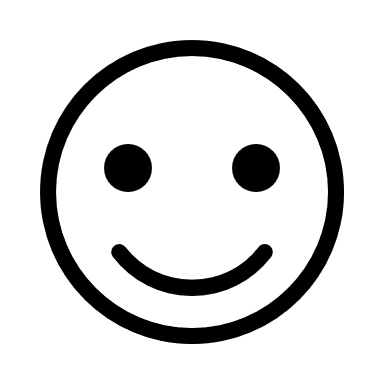 | 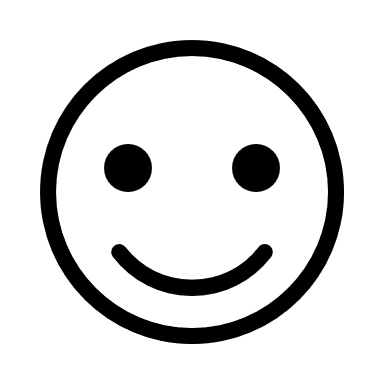 | 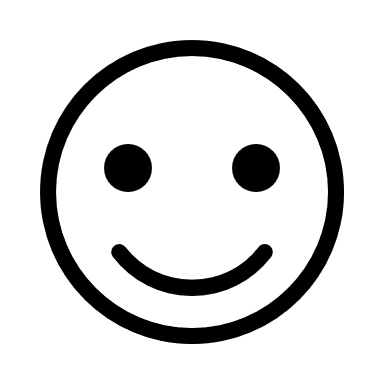 |
| **Diez et al [6]** | 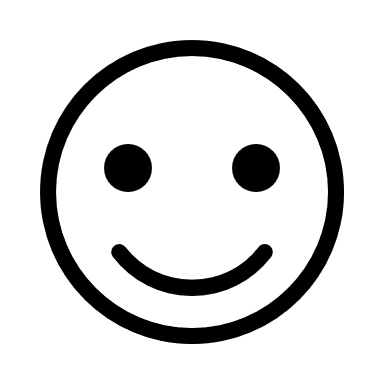 | 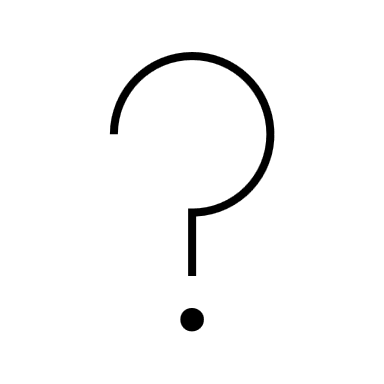 | 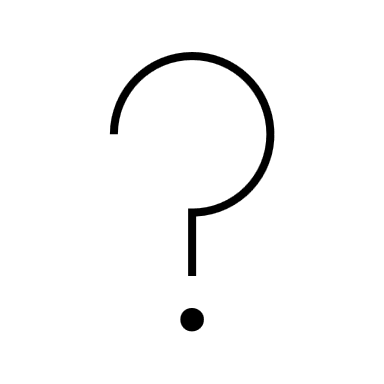 | 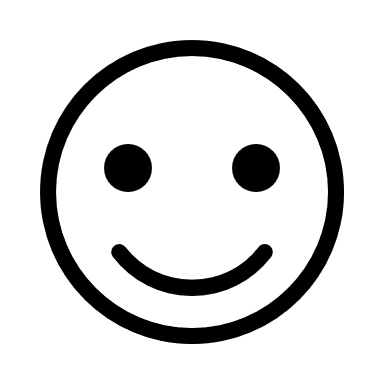 | 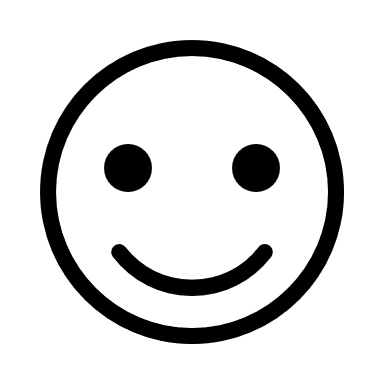 | 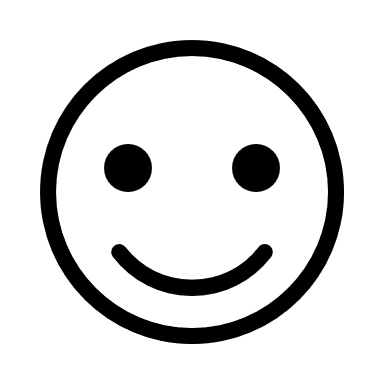 | 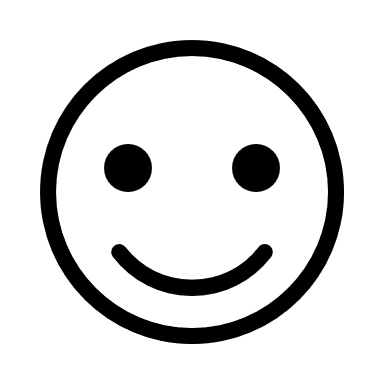 |
| **Wahba et al [20]** | 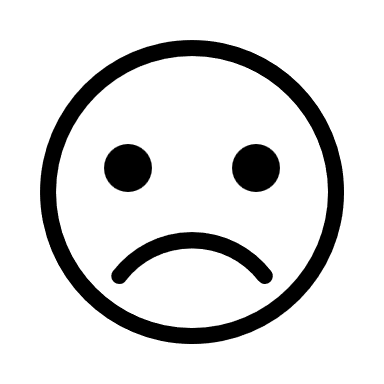 | 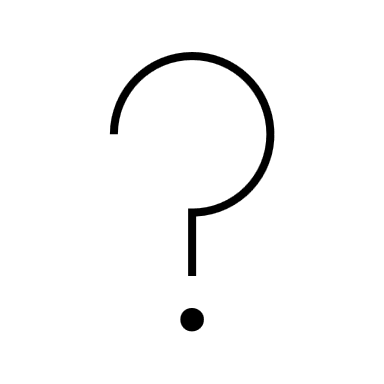 | 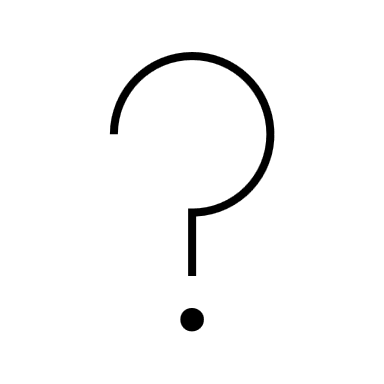 | 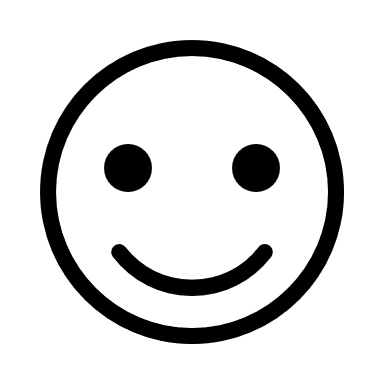 | 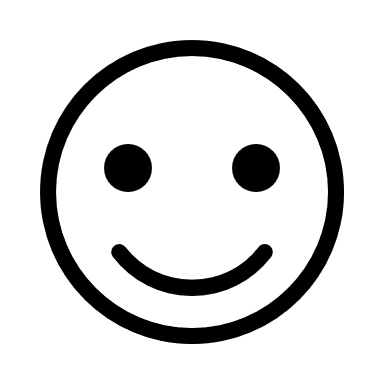 | 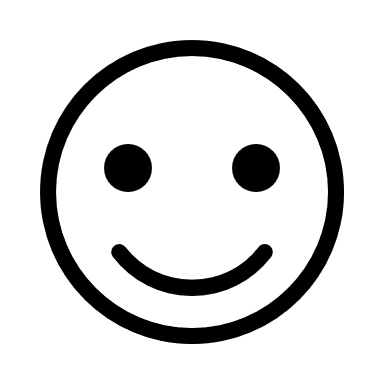 | 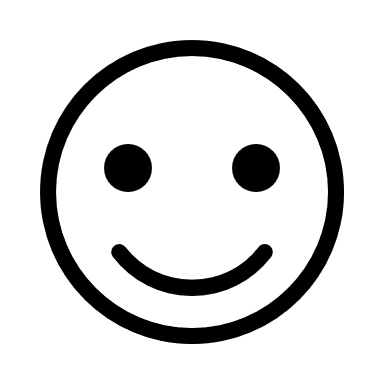 |
| **Weinberger et al [4]** | 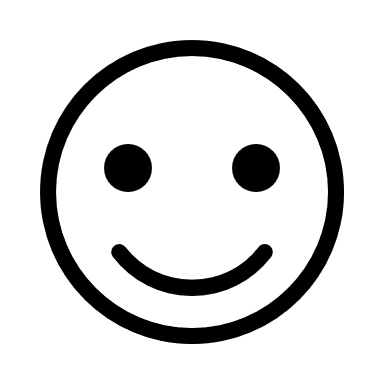 | 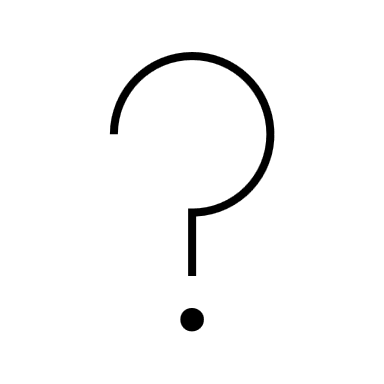 | 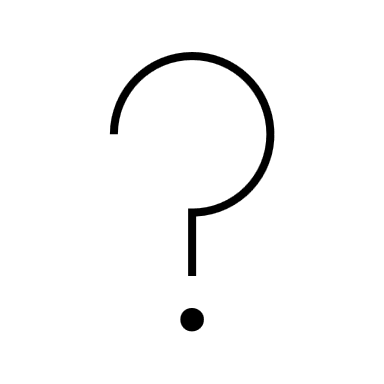 | 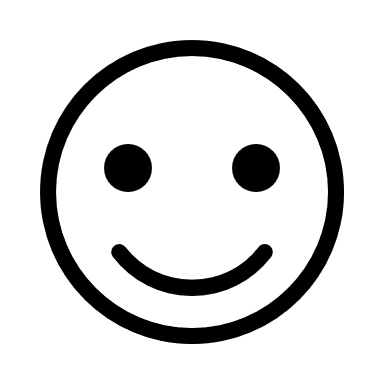 | 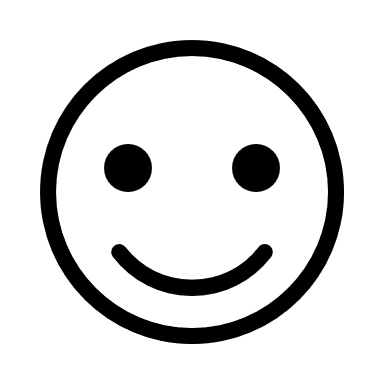 | 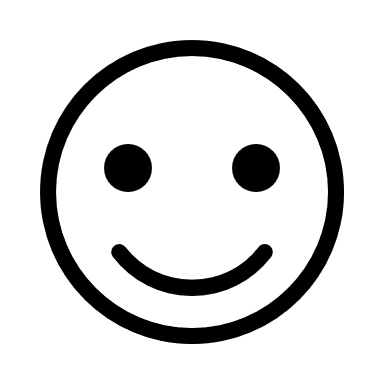 | 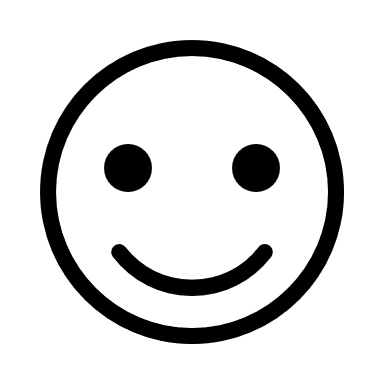 |
| **Yanishi et al [19]** | 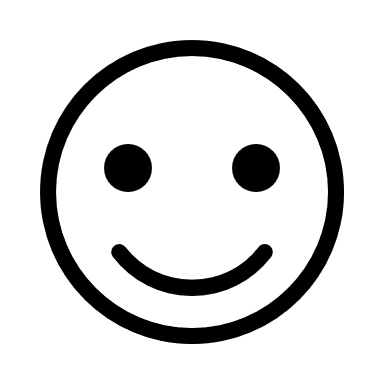 | 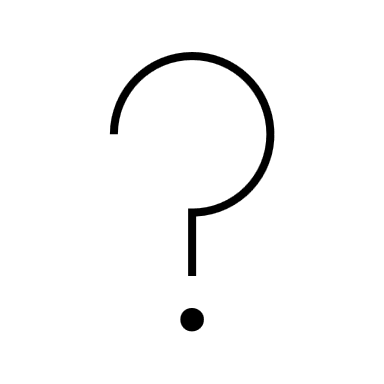 | 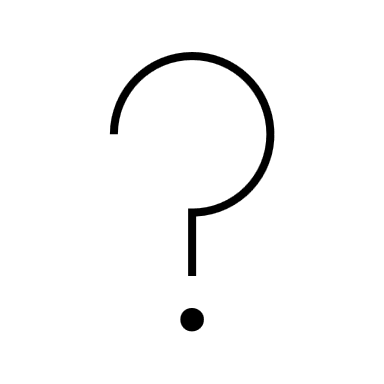 | 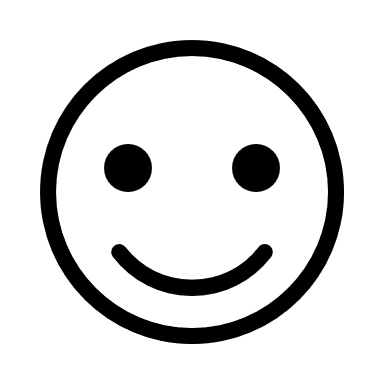 | 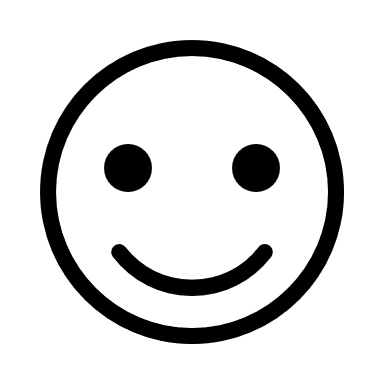 | 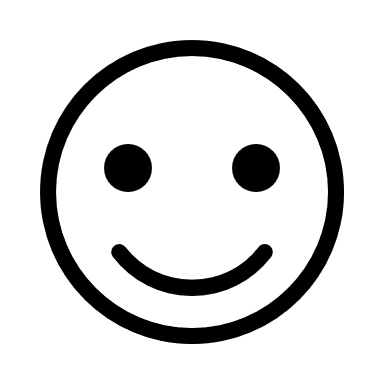 | 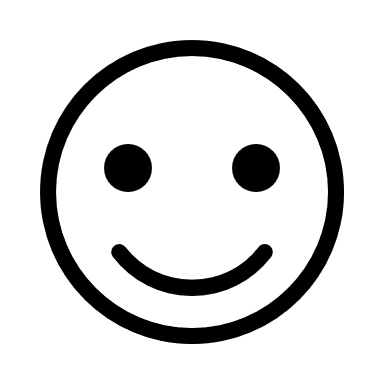 |
| **Yokoyama et al [22]** | 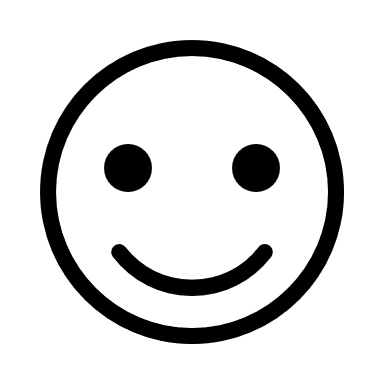 | 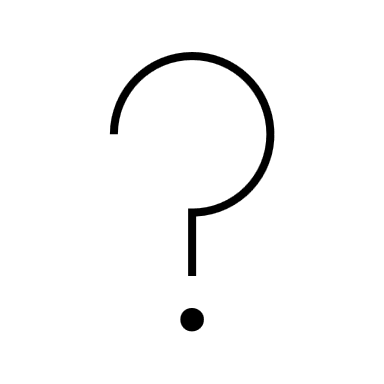 | 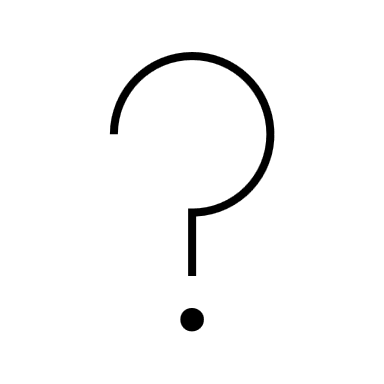 | 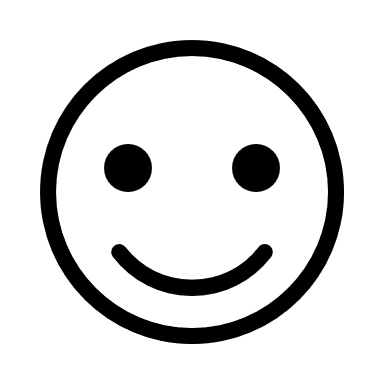 | 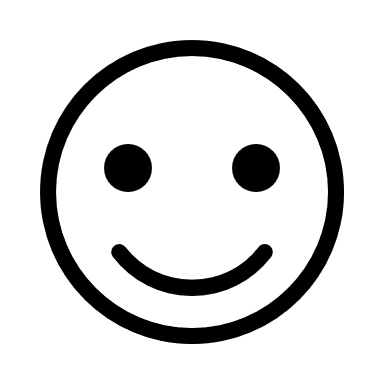 | 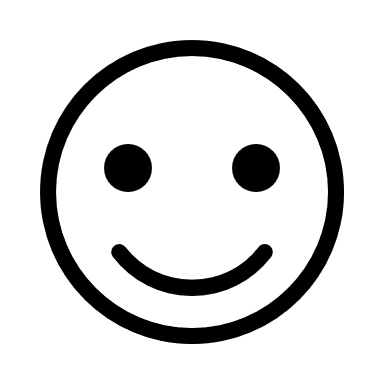 | 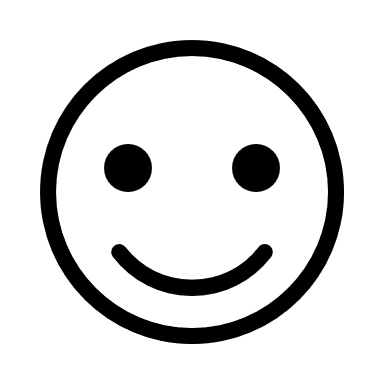 |
| **Barbas et al [23]** | 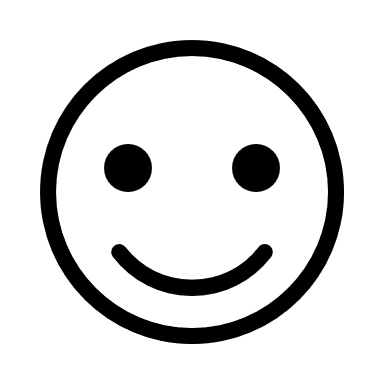 | 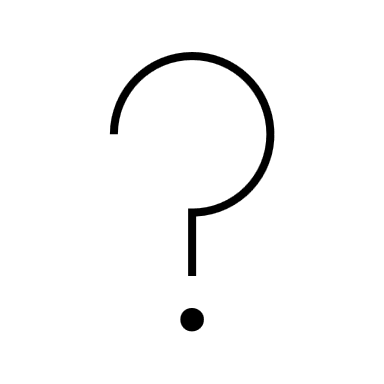 | 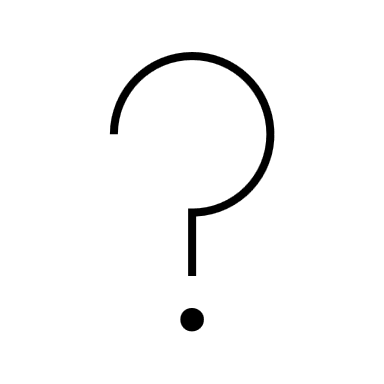 | 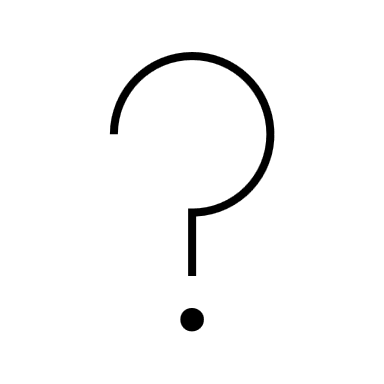 | 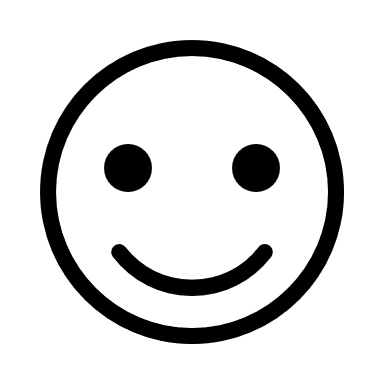 | 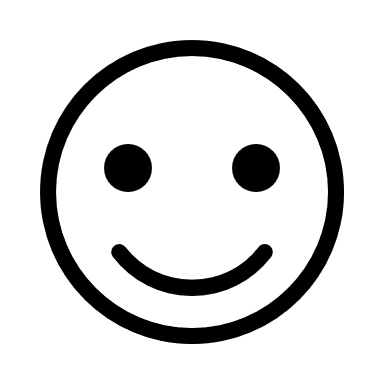 | 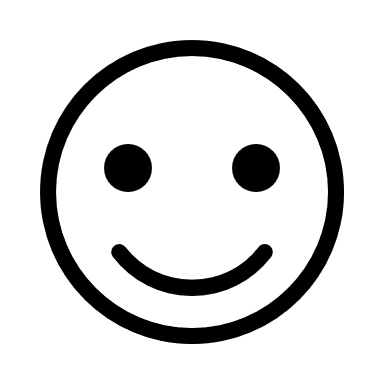 |
| **Lee et al [17]** | 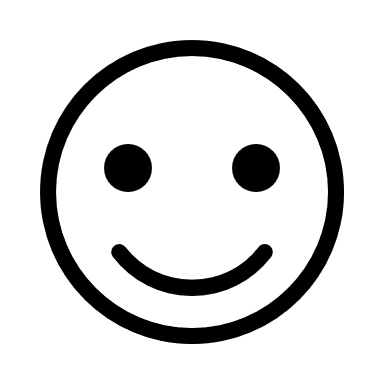 | 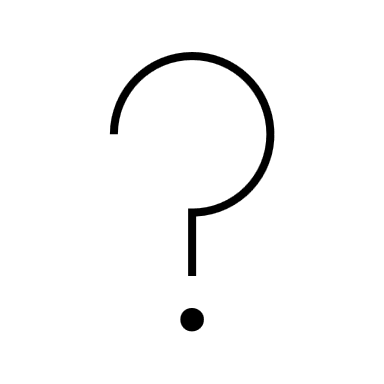 | 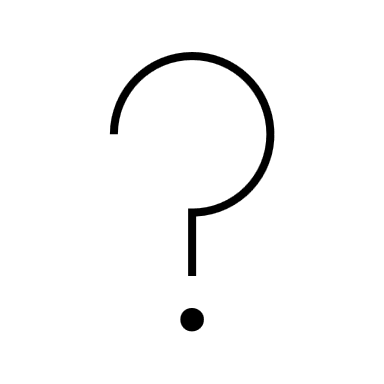 | 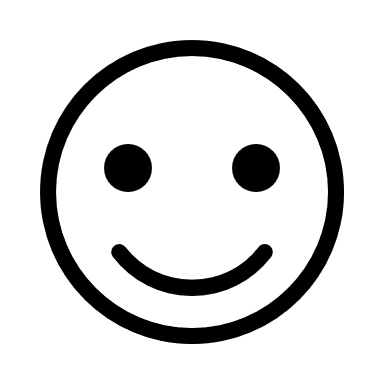 | 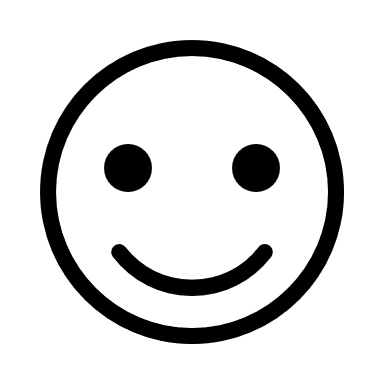 | 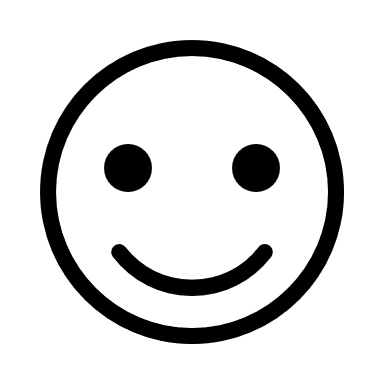 | 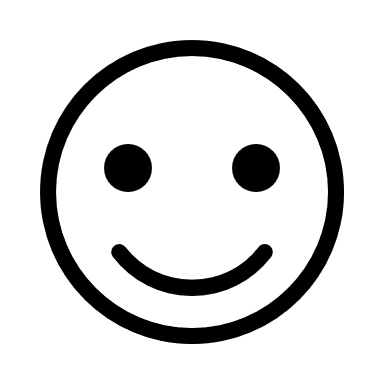 |
| **Mitsui et al [16]** | 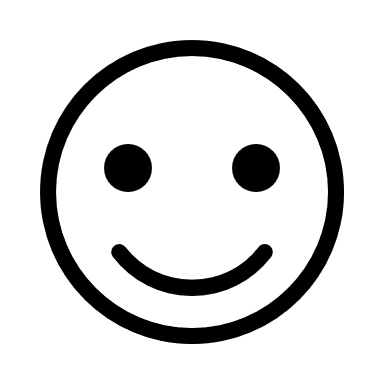 | 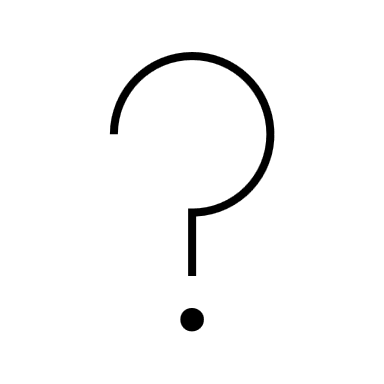 |  |  |  |  |  |
| **Lange et al [15]** |  |  |  |  |  |  |  |
| **Harper et al [25]** |  |  |  |  |  |  |  |
| **Lal et al [11]** |  |  |  |  |  |  |  |
| **Krumm et al [3]** |  |  |  |  |  |  |  |
| **Hun Eum et al [13]** |  |  |  |  |  |  |  |
| **Almeida et al [18]** |  |  |  |  |  |  |  |

|  | **Low risk** |
| --- | --- |
|  | **Unclear** |
|  | **High risk** |
