# Supplementary material for: Can CT or MRI volumetry substitute scintigraphy in living kidney donor evaluation? A systematic review
Source: World J Urol. 2024 Jun 21;42(1):382. doi: 10.1007/s00345-024-05024-y (PMC11192666; doi:10.1007/s00345-024-05024-y)
Supplement: Supplementary file 2 — Supplementary file2 (DOCX 17 KB) [file 345_2024_5024_MOESM2_ESM.docx]

**Appendix 1. Search strategy**

1. **Search strategy for the MEDLINE database**
2. **Concept of living donor**

**Keywords:** living donor

**Search:** (living OR donor OR donation OR “living donors”[Mesh])

1. **Concept of renal function**

**Keywords:** renal function, glomerular filtration rate, kidney injury, renal insufficiency

**Search:** (“Renal Function*”[tiab] OR “kidney function*”[tiab] OR Glomerular Filtration Rate OR "Acute Kidney Injury"[Mesh] OR chronic renal insufficiency OR “glomerulus filtration rate”[tiab])

1. **Concept of image test (MRI or CT)**

**Keywords**: CT volumetry, MRI volumetry, scintigraphy

**Search**: (ct angiography OR ((renal OR kidney) AND (scintigraphy OR Scintiphotography OR CT volumetry OR computer tomographic volumetry)) OR renography) OR (MRI OR ((renal OR kidney) AND (scintigraphy OR Scintiphotography OR MRI volumetry OR magnetic resonance volumetry)) OR renography)

**Search:** (((living OR donor OR donation OR "living donors"[Mesh])) AND (("Renal Function*"[tiab] OR "kidney function*"[tiab] OR Glomerular Filtration Rate OR "Acute Kidney Injury"[Mesh] OR chronic renal insufficiency OR "glomerulus filtration rate"[tiab]))) AND ((ct angiography OR ((renal OR kidney) AND (scintigraphy OR Scintiphotography OR CT volumetry OR computer tomographic volumetry)) OR renography) OR (MRI OR ((renal OR kidney) AND (scintigraphy OR Scintiphotography OR MRI volumetry OR magnetic resonance volumetry)) OR renography))

Total: 349

1. **Search strategy for the EMBASE database**

**Search:** ('living donor'/syn OR 'living donor*') AND ('kidney function'/syn OR 'kidney function*' OR 'acute kidney failure'/syn OR 'acute kidney failure' OR 'chronic kidney failure'/syn OR 'chronic kidney failure' OR 'glomerulus filtration rate'/syn OR 'glomerulus filtration rate') AND ('computed tomographic angiography'/syn OR 'computed tomographic angiograph*' OR 'renography'/syn OR 'renograph*' OR ((renal:ti,ab OR kidne*:ti,ab) AND ('ct volumetry':ti,ab OR 'computed tomographic volumetr*':ti,ab)) OR (('mri volumetry' OR 'mri scanner' OR 'nuclear magnetic resonance' OR 'nuclear magnetic resonance imaging') AND (renal:ti,ab OR kidne*:ti,ab)))

Total: 287

1. **Search strategy for the COCHRANE database**

ID Search Hits

Search Hits

#1 MeSH descriptor: [Living Donors] explode all trees 1004

#2 ((living OR donor* OR donat*)):ti,ab,kw 54532

#3 #1 OR #2 54606

#4 ("Renal Function*" OR "kidney function*" OR "glomerular filtration rate" OR "glomerulus filtration rate" OR "chronic renal insufficiency" OR "Acute Kidney Injury"):ti,ab,kw 33195

#5 MeSH descriptor: [Glomerular Filtration Rate] explode all trees 12460

#6 MeSH descriptor: [Acute Kidney Injury] explode all trees 5681

#7 MeSH descriptor: [Kidney Failure, Chronic] explode all trees 13582

#8 #4 OR #5 OR #6 OR #7 42597

#9 ("computed tomography angiography" OR "ct angiography"):ti,ab,kw 1816

#10 MeSH descriptor: [Computed Tomography Angiography] explode all trees 408

#11 #9 OR #10 3064

#12 ("magnetic resonance" OR "MRI"):ti,ab,kw 46891

#13 MeSH descriptor: [Magnetic Resonance] explode all trees 36413

#14 #12 OR #13 47891

#15 ((renal OR kidne*) AND (scintigraph* OR Scintiphotograph* OR "CT volumetry" OR "computer tomographic volumetry")):ti,ab,kw 252

#16 (renograph*):ti,ab,kw 143

#17 MeSH descriptor: [Radioisotope Renography] explode all trees 53

#15 #11 OR #14 OR #15 OR #16 OR 17 50841

#16 #3 AND #8 AND #18 59
